# Concurrent non-ketotic hyperglycinemia and propionic acidemia in an eight year old boy

**DOI:** 10.1016/j.ymgmr.2014.04.007

**Published:** 2014-05-22

**Authors:** Paul S. Kruszka, Brian Kirmse, Dina J. Zand, Kristina Cusmano-Ozog, Elaine Spector, Johan L. Van Hove, Kimberly A. Chapman

**Affiliations:** a National Human Genome Research Institute, National Institutes of Health, Bethesda, MD, USA; b Department of Genetics and Metabolism, Children's National Health Center, Washington, DC, USA; c Section of Clinical Genetics and Metabolism, University of Colorado, Denver, CO, USA

**Keywords:** Propionic acidemia, Non-ketotic hyperglycinemia

## Abstract

This is the first reported case of a patient with both non-ketotic hyperglycinemia and propionic acidemia. At 2 years of age, the patient was diagnosed with non-ketotic hyperglycinemia by elevated glycine levels and mutations in the *GLDC* gene (paternal allele: c.1576_1577insC delT and c.1580delGinsCAA; p.S527Tfs*13, and maternal allele: c.1819G>A; p.G607S). At 8 years of age after having been placed on ketogenic diet, he became lethargic and had severe metabolic acidosis with ketonuria. Urine organic acid analysis and plasma acylcarnitine profile were consistent with propionic acidemia. He was found to have an apparently homozygous mutation in the *PCCB* gene: c.49C>A; p.Leu17Met. The patient was also treated with natural protein restriction, carnitine, biotin, and thiamine and had subjective and biochemical improvement.

## 1 Introduction

Despite our effort to classify all of an individual's findings under one genetic or metabolic diagnosis, individuals can have more than one disorder. Whole exome studies have illustrated the possibility of more than one diagnosis within a single complex patient. However, how often do we think about more than one biochemical cause and thus, pursue further evaluations to examine more than one cause explaining findings in a single patient? Here, we present a patient who has non-ketotic hyperglycinemia (NKH) and a delayed diagnosis of propionic acidemia (PA).

Elevations in plasma or serum glycine can be several different disorders including non-ketotic hyperglycinemia or ketotic hyperglycinemia. Non-ketotic hyperglycinemia (NKH) is a neurobiochemical disorder in which the glycine cleavage system (encoded by the genes *GLDC*, *AMT*, and *GCSH*) is unable to function resulting in elevated glycine [1]. Patients with NKH often present in the neonatal period with lethargy and seizures. Furthermore, patients with an attenuated form of NKH can present later in infancy, have variable degree of developmental delays, hyperactivity, and choreatic movements. Individuals with NKH have elevated levels of glycine in plasma and in CSF and have an elevated CSF:plasma glycine ratio. Typically, they do not have ketones in plasma or urine [1].

In contrast, ketotic hyperglycinemia, predominately caused by the organic acidurias, propionic acidemia, (PA, from mutations in the genes *PCCA* and *PCCB*) and the methylmalonic acidurias (MMAs), presents with increased ketones in urine and serum [2]. Individuals with PA have characteristic elevations in the organic acids, 3-hydroxypropionic acid and methylcitrate, but can also have increased plasma glycine levels. These individuals usually present with ketoacidosis and symptoms such as lethargy, vomiting and can develop coma [2], [3]. Seizures can occur, but usually during acidosis episodes [4].

## 2 Case report

Several hints presented throughout the history of this patient indicate that there may be more than one cause of his symptomatology. These included a need for carnitine at one year of life and multiple episodes of metabolic ketoacidosis.

The patient's birth history was unremarkable with normal growth parameters, but he had poor breast feeding early on requiring readmission for dehydration and was started on formula. At one month of age, the patient presented with seizures. He had a normal brain MRI, an EEG with “a left focal seizure focus”, and he was treated with phenobarbital and valproate. By 2 months of age, the infant demonstrated upper extremity extensions, eye rolling, and repetitive mouth movements. These movements did not correlate with seizures on video EEG, although the EEG demonstrated right and left hemispheric sharp and slow waves.

By 1 year of age, the EEG showed hypsarrhythmia and the patient was diagnosed with intractable seizures and epileptic encephalopathy. He was admitted with metabolic acidosis with a low bicarbonate of 2.5 mmol/L, fever, and continued to have seizures. By history, he was also diagnosed with low total and free carnitine levels, and started on supplementation with carnitine. Biochemical testing was reportedly done at this time at another institution, including urine organic acids and they were reportedly not diagnostic.

At 2 years of age, patient was found to have choreiform-like movements. He was diagnosed with NKH after finding elevated glycine levels in plasma and CSF with an elevated ratio of CSF: plasma glycine. The diagnosis was confirmed by identification of two mutations in the *GLDC* gene: paternal: c.1576_1577insC delT and c.1580delGinsCAA; p.S527Tfs*13, and a missense mutation on the maternal allele c.1819G>A; p.G607S. This mutation affects a fully conserved amino acid, is predicted to be deleterious by Polyphen-2 [5], and has been observed in another patient with NKH (Van Hove, unpublished observation).

**Fig. 1:**
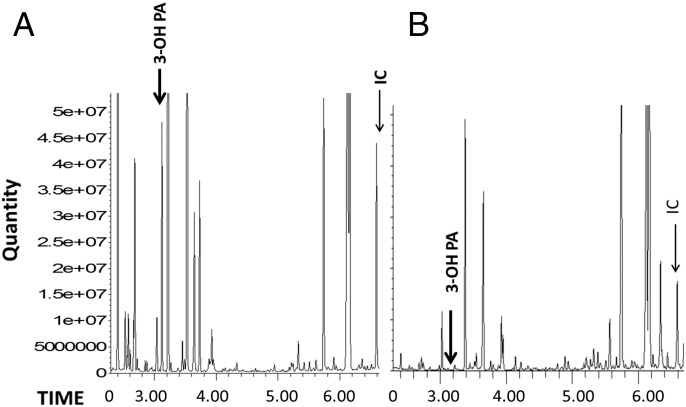
Urine organic acids profiles (Key: 3-OH PA: 3-hydroxypropionic acid, IC: internal standard). Of note our biochemistry laboratory did not quantitate 3-hydroxypropionate at the time the initial spectrum was obtained. A. Marked excretion of 3-hydroxypropionate with normal excretion of methylcitrate while patient is on ketogenic diet. B. Undetectable 3-hydroxypropionic acid after initiation of therapy for PA.

Between the ages of 1 and 8 years, the patient had multiple hospitalizations for metabolic acidosis and ketosis. He made little developmental progress resulting in an inability to ambulate and no functional speech. At 8 years of age after being placed on ketogenic diet for better seizure management, he became lethargic and had severe metabolic acidosis with ketonuria. Urine organic acid analyses were repeated and showed a large peak of 3-hydroxypropionic acid (Fig. 1A), and the acylcarnitine profile revealed elevated propionylcarnitine at 1.2 μmol/L (age normal < 0.88 μmol/L) (Table 1).

The patient was treated with natural protein restriction, carnitine, biotin, and thiamine with resolution of the metabolic acidosis and of the propionate metabolites in the urine organic acids profile (Fig. 1B). Very few differences are apparent in other metabolic measures (Table 1).

The diagnosis of propionic acidemia was confirmed by finding an apparently homozygous mutation in the *PCCB* gene: c.49C>A; p.Leu17Met. There was no normal sequence identified in this sample. This change has been described in one other patient who had an additional two mutations. However, this amino acid is not conserved across species. Our patient's parents deny consanguinity and father was not available for further testing.

For our patient, subjective long term improvement was reported by his parents with increased level of alertness despite very small changes on laboratory. His teachers felt that he made more progress in the one year following addition of treatment for PA, than he had made in the previous 3 years. Moreover, he completed his goals for one year within 4 months requiring a revision of his individual education plan. Therefore, addition of therapy for PA improved his well-being and further supports the diagnosis of PA in addition to NKH.

Tracheostomy was placed for central and obstructive apnea at 9 1/2 years. Unfortunately, our patient passed away at home 1 week after the procedure of an unknown cause. He was biochemically stable at discharge.

## 3 Discussion

This patient presented with attenuated classic non-ketotic hyperglycinemia which was late onset, should lead to mild developmental delay, a seizure disorder and chorea since it was caused by a typically mild missense mutation identified in *GLDC*. However, unusual for a patient diagnosed with NKH, he had a number of hospitalizations with metabolic acidosis and ketonuria, indicating a more complex problem. Moreover, he was severely delayed with no functional speech and lack of ambulation.

The institution of the metabolic stress of a ketogenic diet resulted in metabolic decompensation resulting in the identification of abnormal excretion of urine organic acids typical of propionic acidemia, such as 3-hydroxypropionic acid. The dysfunction of the propionyl-CoA carboxylase in this patient was mild and pre- and post-ketogenic diet urine organic acid analyses were not always abnormal (Fig. 1, Table 1), eluding the identification except for when there was significant metabolic stress (i.e., ketogenic diet). Additionally, the finding of hyperglycinemia was attributed to NKH negating further evaluation. Other indicators for additional diagnoses in this patient's history included carnitine deficiency and multiple episodes of ketonuria and acidosis, which should have prompted the workup that led to the second diagnosis in this patient sooner.

To our knowledge this is the first case of combined PA and NKH in the same patient. We can only hypothesize that the change seen in *PCCB* is mild and may not have manifested in the absence of NKH. The elevated glycine seen in PA is purposed to be caused by inhibition of the glycine cleavage system by elevations in propionyl CoA. Of note, related to this case, there are no reports of the PCC enzyme directly interacting with the glycine cleavage system. However, both are located in the inner mitochondrial membrane and one could suppose that mutations, even otherwise very mild ones, in both *GLDC* and *PCCB* might affect the inner membrane surface proteins' structure and thus, their function. This may be an explanation for the very mild biochemical phenotype seen, yet the significant improvement observed with therapies. Unfortunately, this patient passed away unexpectedly so we do not have samples from him to test this hypothesis.

Identification of and treatment for PA greatly improved this patient's and his family's quality of life. Thus, when all symptoms cannot be explained by a single diagnosis, one must consider a more complex problem, in this case the co-existence of both non-ketotic and ketotic hyperglycinemia. Although the availability of genetic testing is expanding, good biochemical phenotype will continue to be important to diagnosing and adequate treating our patients. Furthermore, patients, such as our patient, may help us understand the pathophysiology of these diseases more thoroughly, if we only consider the possibility.

## Figures and Tables

**Table 1:** Selected biochemical laboratory results of our patient on ketogenic and following initiation of treatment for PA as well as the normal ranges for our laboratory.

Laboratory	Our patient on ketogenic diet	Our patient following initiation of PA treatment	Normal range for our laboratory
Propionylcarnitine (C3) (plasma)	1.2 μmol/L	0.29 μmol/L	< 0.88 μmol/L
2-Methylcitrate (urine)	0.8 mg/g Cr	0.7 mg/g Cr	0.0–8.0 mg/g Cr
Plasma glycine	517 μmol/L	500 μmol/L	91–482 μmol/L
Plasma isoleucine	15 μmol/L	23 μmol/L	22–107 μmol/L
Plasma leucine	40 μmol/L	75 μmol/L	49–180 μmol/L
Plasma valine	101 μmol/L	101 μmol/L	74–321 μmol/L
